# Altered brain and gut responses to corticotropin-releasing hormone (CRH) in patients with irritable bowel syndrome

**DOI:** 10.1038/s41598-017-09635-x

**Published:** 2017-09-29

**Authors:** Michiko Kano, Tomohiko Muratsubaki, Lukas Van Oudenhove, Joe Morishita, Makoto Yoshizawa, Keiji Kohno, Mao Yagihashi, Yukari Tanaka, Shunji Mugikura, Patrick Dupont, Huynh Giao Ly, Kei Takase, Motoyori Kanazawa, Shin Fukudo

**Affiliations:** 1 Frontier Research Institute for Interdisciplinary Sciences (FRIS), Tohoku University, Sendai, Japan; 2 Behavioral Medicine, Graduate School of Medicine, Tohoku University, Sendai, Japan; 3 Laboratory for Brain-Gut Axis Studies (LaBGAS), Translational Research Center for Gastrointestinal Disorders (TARGID), University of Leuven, Leuven, Belgium; 4 Research Division on Advanced Information Technology, Cyberscience Center, Tohoku University, Sendai, Japan; 5 Department of Integrative Genomics, Tohoku Medical Megabank Organization, Tohoku University, Sendai, Japan; 6 Diagnostic Radiology, Tohoku University Hospital, Sendai, Japan; 7 Laboratory for Cognitive Neurology, University of Leuven, Leuven, Belgium

## Abstract

Stress is a known trigger of irritable bowel syndrome (IBS) and exacerbates its gastrointestinal symptoms. However, underlying the physiological mechanism remains unknown. Here, we investigated hypothalamic–pituitary–adrenal (HPA) axis, colonic motility, and autonomic responses to corticotropin-releasing hormone (CRH) administration as well as brain activity alterations in IBS. The study included 28 IBS patients and 34 age and sex-matched healthy control subjects. IBS patients demonstrated greater adrenocorticotropic hormone (ACTH) responses to CRH than control subjects. Male IBS patients had greater increases in colonic motility than male HCs after CRH. Female IBS patients showed altered sympathovagal balance and lower basal parasympathetic tone relative to female control subjects. Brain responses to rectal distention were measured in the same subjects using functional magnetic resonance imaging, and their associations with individual ACTH responses to CRH were tested. A negative association between ACTH response to CRH and activity in the pregenual anterior cingulate cortex (pACC) during rectal distention was identified in controls but not in IBS patients. Impaired top-down inhibitory input from the pregenual ACC to the HPA axis may lead to altered neuroendocrine and gastrointestinal responses to CRH. Centrally acting treatments may dampen the stress induced physical symptoms in IBS.

## Introduction

Irritable bowel syndrome (IBS) is characterized by idiopathic, chronic recurrent abdominal pain associated with altered bowel habits^1^. Stress is a known trigger of IBS; for example, stressful life events and gastrointestinal symptom exacerbations are correlated in patients with IBS^2,3^. Psychological stress also increases colonic contractions in patients with IBS^4^. Given this influence of stress on IBS, a conceptualization of IBS as a disorder of brain–gut interactions has been adopted both clinically and scientifically^5,6^.

Corticotropin-releasing hormone (CRH) is a key mediator of the stress response, both in the brain and in the gut^7,8^. The hypothalamic paraventricular nucleus governs the neuroendocrine stress response via the hypothalamic-pituitary-adrenal (HPA) axis^8–10^. CRH also acts outside the HPA axis in the central nucleus of the amygdala and bed nucleus of the stria terminalis, through which it regulates adaptive stress responses to both physiological and psychological stimuli^9,10^. CRH-positive neuronal populations have also been identified in the lateral hypothalamus, prefrontal cortex (PFC), anterior cingulate cortex (ACC), and hippocampus^9^. The hippocampus inhibits stress-induced HPA activation via glucocorticoid receptor-mediated negative feedback^11^, and the medial (m)PFC and adjacent pregenual (p)ACC, via connections with the hippocampus, amygdala, hypothalamus, and brainstem, execute top-down modulation of the CRH system^12^. It has been proposed that IBS is associated with autonomic and HPA axis imbalances related to a disturbed balance in prefrontal-amygdala activity^1^.

CRH-positive neurons in the parvocellular paraventricular nucleus and Barrington’s nucleus have functional (albeit not physical) connections with the colon, comprising part of the brain-gut pathway^13–15^. Through brainstem and spinal cord projections, CRH neurons provide input to the catecholaminergic neurons that project to the forebrain and sacral parasympathetic nervous system, which in turn innervate the descending colon^14,15^, and thus mediate stress-related actions on colonic motor function independently of HPA axis activation^4,7^. CRH ligands and receptors are also present in various mammalian peripheral tissues including the gastrointestinal tract and heart^7^; in fact, intracerebroventricular CRH increases heart rate and other cardiovascular activity in a manner similar to stress^7^. Intravenously, however, CRH acts via CRH2 receptors to causes vasodilation and concomitant compensatory tachycardia^16^.

Exogenous CRH administration produces IBS-like features in animals, including anxiety behaviors, hyperalgesia to colorectal distention, increased colonic motility, watery stool/diarrhea, and increased colonic mucosal permeability^4,7^. Furthermore, CRH-mediated colonic hypermotility and hyper-responsiveness of the HPA axis have been reported in patients with IBS^17–19^, though possible sex differences are also involved^19^. Together, these data suggest that dysregulation of the CRH system is a pathophysiological mechanism of IBS. However, studies investigating the brain mechanism underlying HPA axis hyper-responsiveness have not been performed. Therefore, we evaluated patients with IBS and age-matched controls after peripheral administration of CRH, determining HPA axis responsiveness, colonic motility, and autonomic reactivity; we then conducted a brain imaging (functional magnetic resonance imaging; fMRI) study during rectal balloon distention in both groups. We tested the hypothesis that rectal distention-induced brain activity in HPA axis-regulating cortical regions including the medial PFC, ACC, hippocampus, and amygdala correlates with HPA axis reactivity as assessed by ACTH responsiveness to CRH. Of note ACTH responsiveness assessed under no distention condition. We also examined whether intravenous CRH administration increases ACTH and cortisol responses, exaggerates colonic motility, and alters sympathovagal balance in patients with IBS compared to healthy control subjects, taking into account the putative sex differences.

## Results

### Subject characteristics

The study included 28 IBS patients (50% female) and 34 age-matched healthy control subjects (47% female). There were no significant differences in age (p = 0.68) or sex ratio (p = 0.81) between the IBS and control groups. In the IBS group, the mean disease duration was 95.6 ± 66.9 months. IBS patients scored significantly higher level of gastrointestinal symptom-specific anxiety (The Visceral Sensitivity Index: VSI, p < 0.0001), anxiety sensitivity (The Anxiety Sensitivity Index: ASI, p = 0.006). Higher ratio of childhood trauma was found in IBS group (p = 0.006). Regarding IBS severity, IBS patients reported greater severity of abdominal pain (p < 0.0001), duration of abdominal pain (p < 0.0001), severity of abdominal distention (p = 0.0001), and dissatisfaction with bowel habits (p = 0.0013) as well as a lower quality of life (p < 0.0001) than healthy control subjects. These data are summarized in Table 1.

**Table 1:** Subject Characteristics.

	Controls (n = 34)	IBS (n = 28)	*p*
Age	22.2 ± 2.7	21.9 ± 2.7	0.68
Sex (male/female)	(18/16)	(14/14)	0.81
Disease duration (month)		95.6 ± 66.9	
SDS	36.24 ± 6.4	38 ± 8.9	0.38
STAI trait	36.5 ± 1.1	42.7 ± 2.3	0.05
ASI	7.45 ± 4.4	12.32 ± 7.9	0.006*
VSI	3.1 ± 0.9	22.9 ± 2.5	<0.0001*
Childhood trauma (number)			
Total	1 (3%)	8 (28%)	0.006*
Sexual	0	4 (14%)	
Physical	1 (3%)	7 (25%)	
IBS-SI			
Severity of abdominal pain	11.75 ± 23.2	51.1 ± 13.9	<0.0001*
Duration of abdominal pain	0.35 ± 0.5	3.5 ± 1.5	<0.0001*
Severity of abdominal distention	5.5 ± 14.9	31.7 ± 28.3	0.0002*
Dissatisfaction with bowel habits	26.75 ± 29.8	48.7 ± 23.0	0.0021*
Quality of life	7.15 ± 11.7	45.5 ± 24.1	<0.0001*
Overall	51.5 ± 43.9	180.5 ± 54.3	<0.0001*

Data is shown mean ± SD (standard deviation), *Significant differences between groups. Abbreviations: ASI, Anxiety Sensitivity Index; IBSSI, IBS Sensitivity Index; SDS, Self-Rating Depression Scale; STAI, State-Trait Anxiety Inventory; VSI, Visceral Sensitivity Index.

### Effect of CRH administration on HPA axis, colonic motor responses and autonomic function

**Figure 1:**
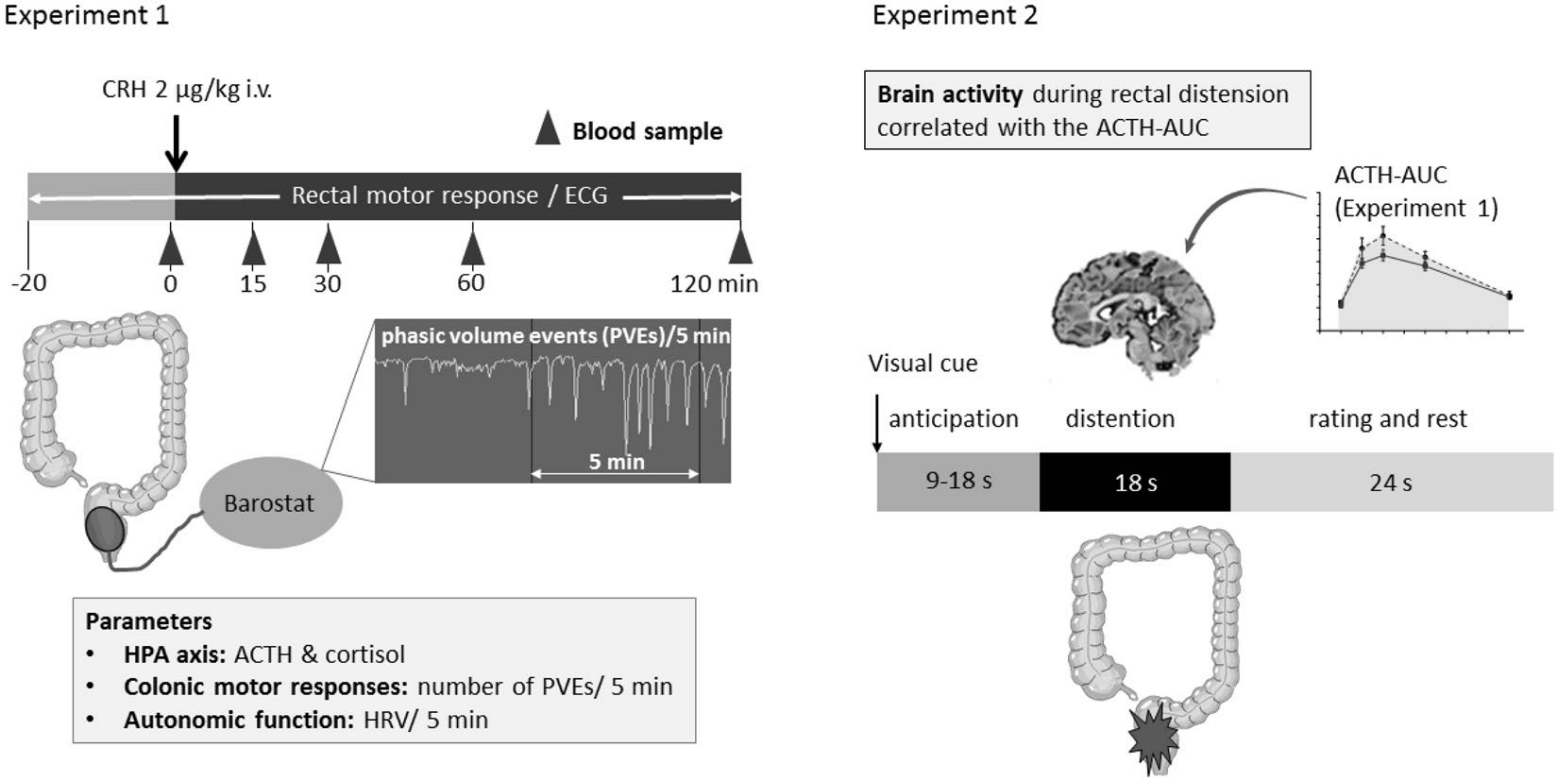
Design of the CRH administration (experiment 1) and fMRI during rectal distention (experiment 2) tests. Abbreviations: ACTH, adrenocorticotropic hormone; AUC, area under the curve; CRH, corticotropin-releasing hormone; ECG, electrocardiogram; HPA axis, hypothalamic-pituitary-adrenal axis; HRV, heart rate variability; PVEs, phasic volume events.

The study comprised of two experiments (Fig. 1). In the first experiment, plasma adrenocorticotropic hormone (ACTH) and cortisol, colorectal phasic volume events^20^, and electrocardiography (ECG) were measured from 20 min before until 120 min after intravenous CRH administration (2 μg/kg).

#### ACTH and cortisol

**Figure 2:**
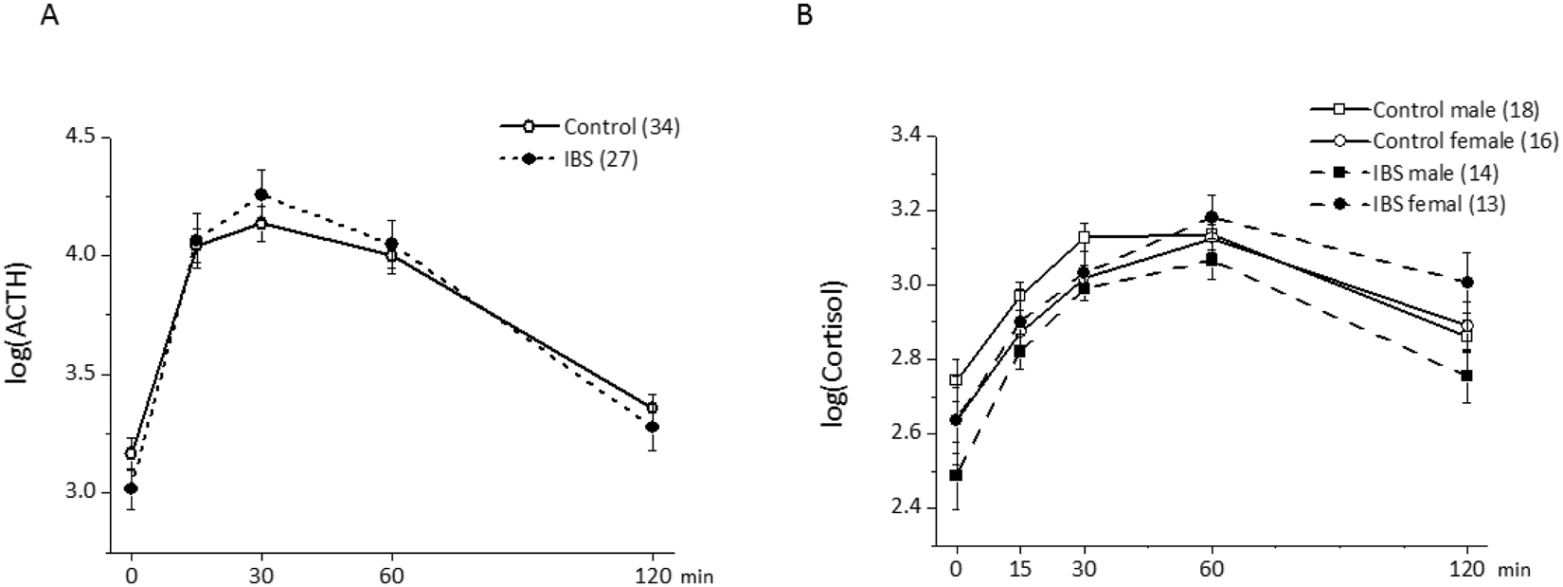
(**A**) ACTH responses to intravenous CRH administration in patients with IBS and healthy control subjects. (**B**) Cortisol responses to intravenous CRH administration in patients with IBS and healthy control subjects. Abbreviations: ACTH, adrenocorticotropic hormone; CRH, corticotropin-releasing hormone; IBS, irritable bowel syndrome.

Blood samples for ACTH and cortisol assays were drawn immediately before and 15, 30, 60, and 120 min after CRH injection. After eliminating non-significant interaction effects, the final model for ACTH included a significant main effect of time (F_(4, 236)_ = 176.46, p < 0.0001), and a significant group (IBS versus control)-by-time interaction effect (F_(4, 236)_ = 4.31, p = 0.0026) (Fig. 2A). The main effects of group (F_(1, 58)_ = 0.03, p = 0.87) and sex (F_(1, 58)_ = 0.89, p = 0.36) were not significant. Planned follow-up contrasts on the significant group-by-time interaction effect showed that the increase in ACTH relative to the pre-infusion time point was significantly larger in patients with IBS compared to healthy control subjects at 30 min after infusion (p_Holm_ = 0.04). Differences at other time points were not significant (all p_Holm_ > 0.15).

After eliminating non-significant interaction effects, the final model for cortisol included a significant main effect of time (F(4,236) = 90.8, p < 0.0001) and significant sex-by-time (F(4,236) = 6.56, p < 0.0001) and group-by-sex (F(1,57) = 4.84, p = 0.019) interaction effects (Fig. 2B). The main effects of group (F(1,57) = 2.52, p = 0.12) and sex (F(1,57) = 1.53, p = 0.22) were not significant. Planned follow-up contrasts for the sex-by-time interaction effect showed that increases in cortisol relative to the pre-infusion time point were not significantly different between sexes at any post-infusion time point (p_Holm_ > 0.83 for all comparisons). For the group-by-sex interaction, analyses according to group showed no sex difference in patients with IBS and control groups (p_Holm_ > 0.07). Separate analyses according to group revealed a significant difference between groups for male subjects (i.e., lower levels of cortisol in male patients with IBS relative to male control subjects) [F(1,57) = 9.59, p_Holm_ = 0.012] but not female subjects (p_Holm_ = 0.59).

Notably, no significant influence of abuse history was found (details in the supplemental material) on ACTH and cortisol response, so abuse history was not included in statistical analyses.

#### Colonic motor responses

**Figure 3:**
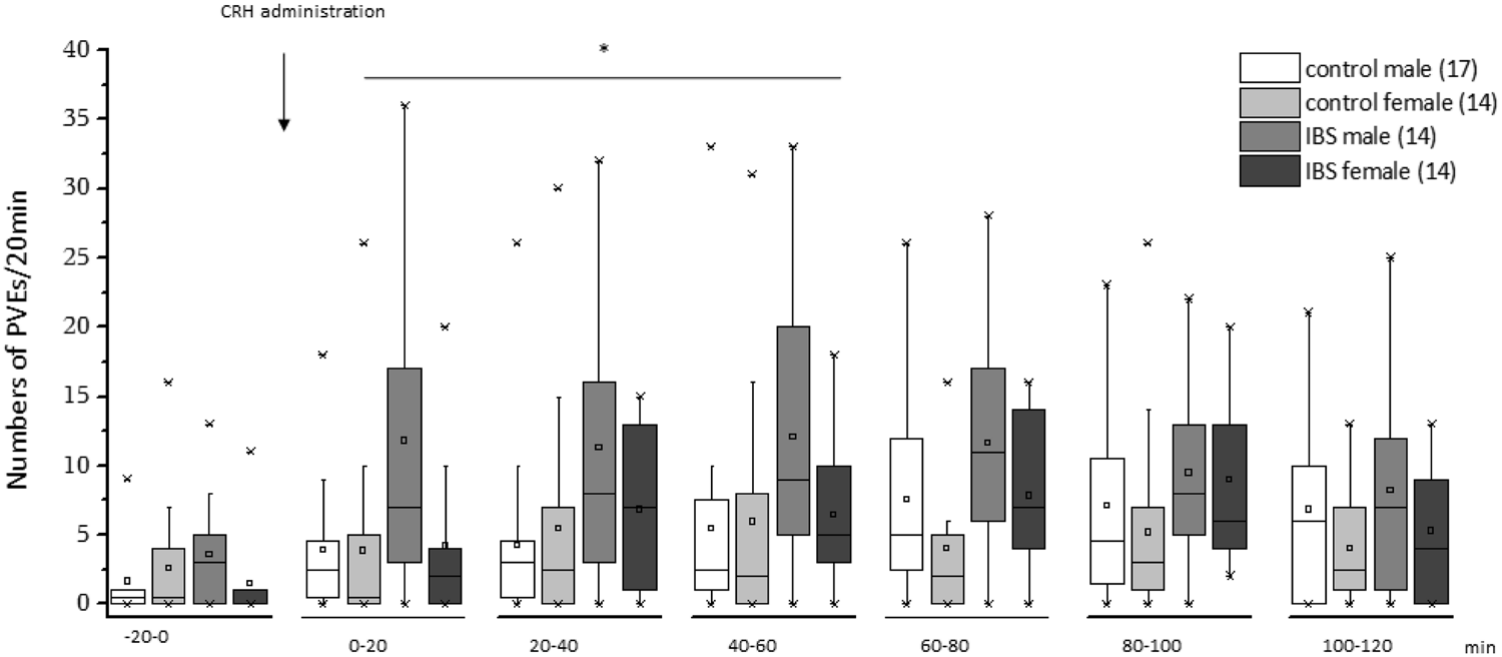
Effect of CRH administration on phasic volume events in patients with IBS and healthy control subjects. The asterisk indicates p = 0.024 for male patients with IBS versus male control subjects from 0–60 min (compared to the pre-infusion baseline (−20-0)). Abbreviations: CRH, corticotropin-releasing hormone; IBS, irritable bowel syndrome.

Number of phasic volume events were measured in 5-min intervals using rectal barostat as index of colonoic motor responses. There was a significant group-by-sex-by-time 3-way interaction effect on phasic volume events (F_(5, 275)_ = 2.63, p = 0.02) (Fig. 3). Separate group analyses revealed a significant main effect of time in the control group (F_(5, 145)_ = 2.41, p < 0.04). Analyses according to sex showed a significant main effect of time in female subjects (F_(5, 130)_ = 3.49, p = 0.005), and a significant group–by-time interaction effect in male subjects (F_(5, 145)_ = 15.38, p = 0.011). Among male subjects, planned contrasts revealed significant between-group differences in the first 3 time points (0–60 min) (p = 0.024), but not the last 3 time points (60–120 min) (p = 0.79). These results indicate that the 3-way interaction effect was mainly driven by the significant difference between male subjects with IBS and controls in the first hour after CRH administration.

#### Autonomic function

**Figure 4:**
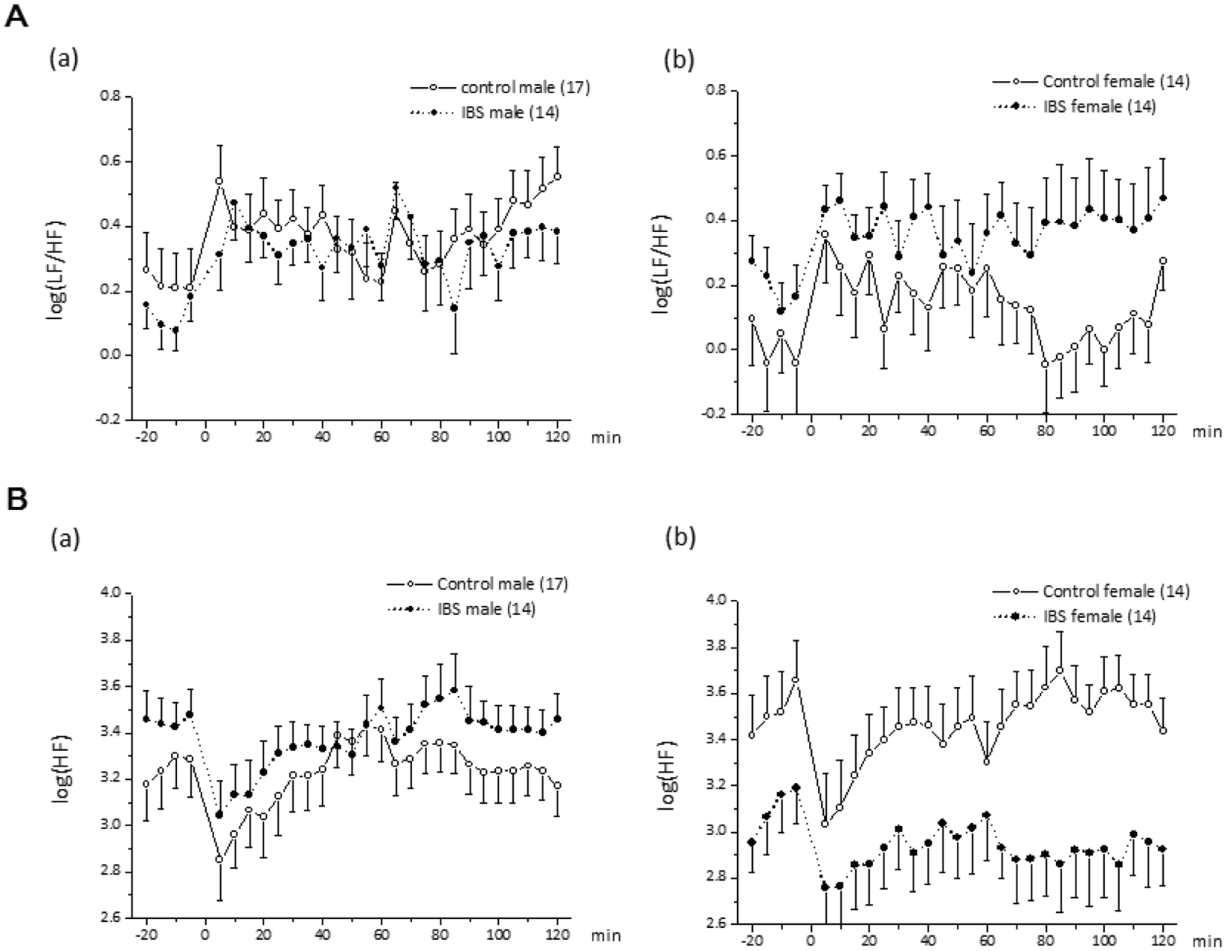
(**A**) Changes in the low frequency/high frequency (LF/HF) band ratio in response to intravenous CRH. (a) Males in the control group versus males in the IBS group. (b) Females in the control group versus females in the IBS group. (**B**) Changes in the high frequency (HF) band in response to intravenous CRH. (a) Males in the control group versus males in the IBS group. (b) Females in the control group versus females in the IBS group. Abbreviations: CRH, corticotropin-releasing hormone; HF, percent high frequency band power; IBS, irritable bowel syndrome; LF, percent low frequency band power.

Heart rate variability (HRV) signal was analyzed in 5 min interval. The percent power in the high frequency (HF) band as a measure of vagal tone, and the low frequency (LF)/HF ratio as an indicator of sympathovagal balance. Regarding the LF/HF ratio used to measure sympathovagal balance, there was a significant group-by-sex-by-time 3-way interaction effect (F_(27, 1390)_ = 1.57, p = 0.033) (Fig. 4A). CRH injection caused an increase in sympathovagal balance over the entire time period over both groups and sexes (all p_Holm_ < 0.004). All other main effects and 2-way interaction effects were non-significant (details not shown). Analyses according to group revealed a significant main effect of time and no significant sex-by-time interaction effect in both groups (detail not shown). In the controls, *post hoc* tests comparing sex in each time bin demonstrated significant differences in the last 2 time bins (p_Holm_ < 0.05 for both comparisons), indicating that LF/HF values in male control subjects did not return to baseline. Analyses by sex revealed that the main effect of group (p = 0.68) and the group-by-time interaction effect (p = 0.53) were not significant in male subjects. In female subjects, however, a significant group-by-time interaction effect (F_(27, 665)_ = 1.52, p = 0.045) was found, but the main effect of group was not significant (p = 0.13). Thus, the 3-way group-by-sex-by-time interaction effect was mainly driven by differences between female subjects in the IBS and control groups, especially towards the end of the measurement period.

When looking at the HF data alone as a marker of vagal tone and after eliminating non-significant interaction effects, the final model showed significant main effects of group (F_(1, 56)_ = 11.21, p = 0.002), and sex (F_(1, 56)_ = 4.83, p = 0.032), with the main effects of group and sex superseded by a significant group-by-sex interaction effect (F_(1, 56)_ = 21.15, p < 0.0001) (Fig. 4B). The group-by-sex interaction effect was driven by a significant effect of sex in patients with IBS (i.e., lower HF was observed across all time points in female subjects) (F_(1, 56)_ = 28.79, p_Holm_ < 0.0001) but not in healthy control subjects (p_Holm_ = 0.25), as well as by a significant group effect in female subjects (i.e., lower HF was observed in the IBS group) (F_(1, 56)_ = 27.91, p_Holm_ < 0.0001) but not in male subjects (p_Holm_ = 0.35).

### Associations with individual ACTH responses to CRH and brain activities

In the second experiment (Fig. 1), brain responses to rectal distention were measured in the same subjects using functional magnetic resonance imaging and associations with individual ACTH responses to CRH were tested.

**Figure 5:**
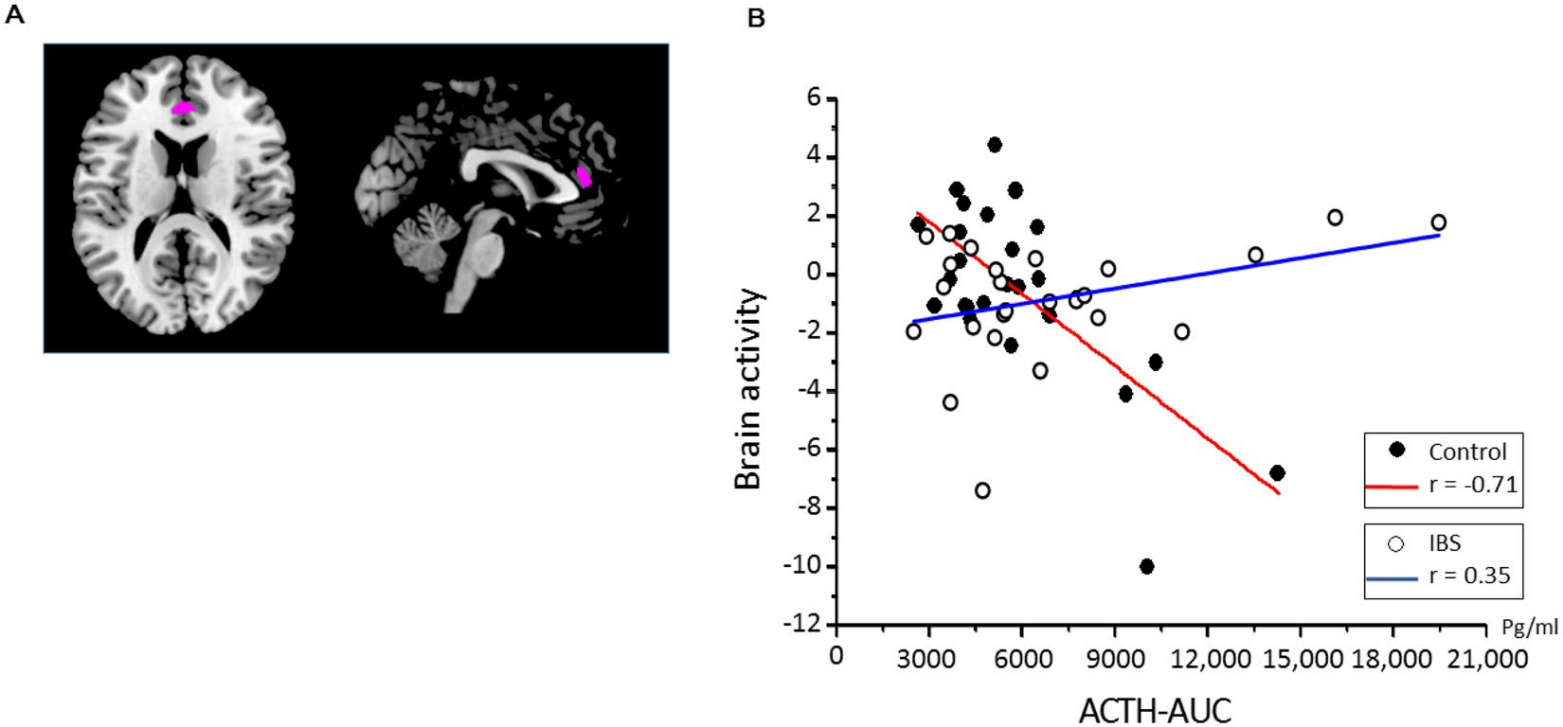
(**A**) Brain activity during rectal distention associated with individual ACTH-AUC responses to CRH between patients with IBS and control subjects. (**B**) Scatter plot and regression of Eigenvariates during rectal distention (versus no distention) and ACTH area under the curve (ACTH-AUC) in patients with IBS versus control subjects for the right anterior cingulate cortex. Abbreviations: ACC, anterior cingulate cortex; ACTH-AUC, adrenocorticotropic hormone-area under the curve; CRH, corticotropin-releasing hormone; IBS, irritable bowel syndrome.

fMRIs acquired during the rectal distention phase demonstrated a significantly stronger negative association between ACTH-AUC (individual area under the curve of the ACTH response to CRH) value and brain response (represented by blood oxygen level-dependent (BOLD) signal). Specifically, such associations were found in the bilateral ACC (right; x = 2, y = 39, z = 14, cluster size = 28, t = 5.4, P_FWE-corrected_ = 0.02, and left, X = −8, y = 38, z = −3, cluster size = 14, t = 5.33, P_FWE-corrected_ = 0.02) and right superior frontal gyrus (x = 21, y = 38, z = 52, cluster size = 15, t = 5.27, P_FWE-corrected_ = 0.03) in the control group relative to the IBS group (Fig. 5A). These results remained significant after controlling for sex and psychological factors as nuisance covariates (Details are provided in the supplemental materials). Figure 5B demonstrates the association between the first Eigenvariate in the right ACC and individual ACTH-AUC values from experiment 1 (see “ACTH and cortisol”). Correlation analysis between the first Eigenvariate and ACTH-AUC revealed that this significant interaction effect was driven by a significant negative association in control subjects (r = −0.701, t = 4.99, p = 0.00004), as well as a non-significant positive association in patients with IBS (r = 0.35, t = 1.77, p = 0.09). Additionally region of interest (ROI) analysis were conducted in HPA axis-regulating cortical regions. The brain response in the right medial PFC, and bilateral pregenual ACC was negatively associated with ACTH-AUC in the control group versus the IBS group, supporting the findings of the whole-brain analysis. ROI analysis revealed a significant negative association between brain signal and ACTH-AUC in the bilateral pregenual ACC and right medial PFC in the control group, whereas there was no significant association in the IBS group (Table 2).

**Table 2:** Results of region of interest (ROI) analysis during rectal distention.

Side	Region	Between group		Within-group			
				Control		IBS	
		Control > IBS	IBS > Control	Positive	Negative	Positive	Negative
R	mPFC	>0.1	0.009*	>0.1	0.047*	>0.1	>0.1
L	mPFC	>0.1	>0.1	>0.1	>0.1	>0.1	>0.1
R	pACC	>0.1	< 0.001*	>0.1	0.018*	0.082	>0.1
L	pACC	>0.1	< 0.001*	>0.1	0.004*	>0.1	>0.1
R	Hippocampus	>0.1	>0.1	>0.1	>0.1	>0.1	>0.1
L	Hippocampus	>0.1	>0.1	>0.1	>0.1	>0.1	>0.1
R	Amygdala	>0.1	>0.1	>0.1	>0.1	>0.1	>0.1
L	Amygdala	>0.1	>0.1	>0.1	>0.1	>0.1	>0.1

Corrected p values are presented and * indicates significant p value, IBS: irritable bowel syndrome; pACC: pregenual anterior cingulate cortex; mPFC: medial prefrontal cortex.

## Discussion

The study is the first to comprehensively assess HPA axis, autonomic, and colonic responses to CRH in IBS and healthy controls and link these responses to brain responses to rectal distension in regions known to be involved in top-down control of the abovementioned peripheral stress responses. First, we found that intravenous CRH produced a larger ACTH response in patients with IBS than in healthy control subjects. Second, CRH induced exaggerated colonic contractions in the first hour in male patients with IBS relative to male control subjects. Third, female patients with IBS demonstrated lower parasympathetic tone than female control subjects both pre- and post-infusion. Fourth, our fMRI results showed that the response of the pregenual ACC to rectal distension was negatively correlated with ACTH-AUC in the control group, but not in the IBS group, resulting in a significant between-group difference for this association. These results suggest impaired top-down inhibitory input from the pregenual ACC to the HPA axis, which may be responsible for the altered neuroendocrine and colonic responses to CRH. Although altered colonic motility, HPA axis response, autonomic function, and brain function in IBS have been reported separately in previous studies, few studies have linked them in a single patient sample. One unique point of the current study is that the abnormal peripheral parameters which may underlie symptoms in IBS were observed with alternation of brain function which may be responsible for the stress response in the same sample population. As previously advocated, IBS is not a disease of only abdominal symptoms or brain alternation, but a disease with an altered physiological stress response system in the brain combined with peripheral interactions. Collectively, our findings indicate that IBS involves CRH-dependent dysregulation of the brain-gut axis. This supports the conceptualization of IBS as a disorder of brain-gut interactions with stress response system, such as ANS and the HPA-axis, serving as an important interface.

In the present study, ACTH reactivity was greater in patients with IBS than in healthy control subjects. Two similar previous reports suggested that HPA axis hyper-reactivity may have been related to increased anterior pituitary CRH1 receptor expression in patients with IBS^17,18^. On the other hand, a recent study reported similar ACTH and cortisol responses to CRH in IBS and controls^19^. The authors suggested the differences may be related to the use of human versus ovine CRH, or to the predominance of IBS-D (in the former 2 studies) versus IBS-C^19^. Our study replicated the results of the former 2 studies using the same experimental conditions, and like those studies, and thus support their evidence indicating disturbed top-down modulation in IBS. Interestingly, cortisol responses showed no group differences; however, cortisol levels in male patients with IBS were already lower at baseline than those in healthy control male subjects. It should be noted that cortisol secretion is not controlled solely by ACTH, though, but is also regulated by direct autonomic adrenal gland innervation^21^.

A clinical study found that intravenous CRH produced greater increases in duodenal motor activity and contractions in the descending colon in patients with IBS than in healthy volunteers^17^, with which our findings are in line. One explanation for this is upregulation of colonic CRH1 receptors^8,17^. CRHR1 mRNA is expressed throughout the gastrointestinal tract, with the highest levels in the human ileum and rectum, and CRH immunoreactive cells are present in submucosal and myenteric neurons of the human colon. In patients with IBS, the non-selective CRH antagonist α-helical CRH attenuated colonic motility induced by rectal transmural electrical stimulation^22^. Peripheral injection of CRH stimulates colonic motility and leads to defecation and watery diarrhea in both rats and mice^7,23^. Taken together with the fact that CRH and α-helical CRH do not cross the blood-brain barrier^24^, these findings suggest that peripheral CRH1 receptor signaling mediates the increased colonic motility and diarrhea in IBS, particularly in males. However, truncal vagotomy, hexamethonium, atropine, and intracerebroventricular astressin (a non-selective CRH antagonist) all abolished peripheral CRH-induced exaggerated colonic motility, suggesting some level of central involvement^25^. These hypotheses do not explain our novel finding of a sex difference in the colonic reaction to CRH, though this too may be a factor in IBS pathophysiology.

Intravenous CRH administration increased sympathovagal balance (LF/HF) across both groups and sexes for the duration of the experiment (2 h post-infusion), and decreased parasympathetic tone (HF) in the early post-infusion period, replicating the results of healthy subjects in a previous clinical study^16^. In our study, CRH shifted sympathovagal balance over time towards sympathetic activity in female patients with IBS more than it did in female control participants; the difference was most pronounced in the last (i.e. second) hour, hinting at an impaired recovery from the CRH-induced increase in sympathovagal balance. A meta-analysis of 11 studies showed that patients with IBS have lower HF band power and a higher LF/HF ratio than control subjects^26^, indicative of autonomic dysregulation. This is in agreement with our data, although parasympathetic tone was significantly different between only female subjects. Moreover, increased LF/HF and decreased HF in the postprandial period was demonstrated in IBS-D^27^. Unbalanced autonomic activity in response to rectosigmoid balloon distention was found to a greater extent in men with IBS than in women with IBS^28^, suggesting that the sex difference found herein may be related to sex differences in autonomic function (or dysfunction).

In our study, ACTH responses to CRH administration were correlated with brain responses to rectal distention, and this association was significantly different between the control and IBS groups. In control subjects, individuals with higher ACTH responses showed lower brain signal brain responses to rectal distension in the pregenual (p)ACC, whereas such an association was not present in patients with IBS. The medial (m)PFC/pACC, hippocampus, amygdala, and brainstem nuclei regulate the HPA axis in a top-down fashion^12^, such that the hippocampus inhibits the HPA axis^29^, whereas the amygdala excites it^30^. Bilateral lesioning of the prelimbic subregions, which correspond to Brodmann area 32, enhances ACTH and corticosterone responses and increases c-Fos expression in the PVN following stress in rodents^31^. Thus, the mPFC/pACC, prelimbic dorsal areas appear to exert inhibitory influences on the HPA axis. Importantly, neither dorsal nor ventral mPFC/pACC lesions affect basal ACTH or corticosterone levels^31^, indicating that the mPFC/pACC selectively modulates stress-induced HPA activity^31^.

Human neuroimaging studies have demonstrated a negative association between mPFC/pACC activity and HPA axis-related outcomes. Decreased activity in the orbitofrontal PFC has been associated with increased cortisol secretion in response to psychological or psychosocial stress in several studies^32–34^. In one fMRI study, the degree of deactivation in healthy subjects was linearly correlated with cortisol release in response to the metal stress; however, activation of the pACC was decreased in cortisol responders and increased in non-responders^32^. These data indicate that mPFC/pACC activity may represent individual differences in inhibitory control of the HPA axis in response to stressful condition. This hypothesis is supported by the results of a PET study of combat veterans with and without post-traumatic stress disorder during a trauma recall script viewing, where ACTH responders demonstrated deactivation in the mPFC/pACC compared to ACTH non-responders^35^. In addition, patients with frontal lesions have been reported to exhibit increased HPA axis output^36^. Taken together with our data, mPFC/pACC may play an important role in top-down inhibitory regulation on HPA axis response.

Finally, the function of the mPFC/pACC is altered under chronic stress conditions^31^. Of note, the stress-inhibitory influence of the mPFC/pACC on the paraventricular nucleus is not due to direct innervation^37^. Rather, it is mediated by excitatory glutamatergic and inhibitory GABAergic neurons (e.g., through intermediary network loci in the bed nucleus of the stria terminalis)^37^. Glutamate in the PFC may play a role in the neurochemical response to repeated stress: extracellular levels of glutamate in the mPFC decrease with repeated exposure to tail pinch stress in rodents^38^. This suggests that increased CRH release in IBS, possibly due to chronic-stress related dysfunction of the mPFC/pACC inhibitory system, induces upregulation and/or overactivation of CRH1 receptors in the pituitary gland, similarly to the mechanism of CRH hypersensitivity in the gut (i.e., excessive ACTH secretion).

The current study had several limitations. First, our sample size is relatively small. Further, we did not control for the menstrual cycle in female subjects for reasons of practicality (scheduling and equipment availability issues). Considering the interaction of the HPA axis with circadian rhythms, the fact that the examination took place in the morning may have influenced our results. However, it was difficult for subjects to fast until the afternoon, so examinations were conducted in the morning for ethical reasons. Although blood pressure may change after CRH administration and influences the ANS response^39^, it was not measured due to the possible influence blood pressure measurement with cuff inflation in the arm on endocrine responses. Substantial inter-study variation has been indicated in brain-imaging studies^40^ and reproducibility of our brain-imaging results is warranted in future studies. In IBS patients, bile acid malabsorption was not screened although it can produce IBS-D like symptoms^41^. The IBS patients in the present study consist of 24 IBS-diarrhea and 4 IBS-mixed (non-IBS-constipation). The sample size is not enough to demonstrate of these two group in this study. These limitations are need to solve and confirm in large population in future studies. Despite these limitations, we believe that the current study provides important and novel findings regarding brain–gut interactions in IBS.

Intravenous CRH administration produced a larger ACTH response in IBS subjects than in healthy control subjects, and increased colonic motility was noted in male IBS subjects. Further, female IBS subjects exhibited altered sympathovagal balance responses to CRH, and a lower basal parasympathetic tone relative to female control subjects. Lastly, control subjects, but not patients with IBS, showed an association between the ACTH response to CRH infusion and mPFC/pACC activity evoked by rectal distention. These results suggest that mPFC/pACC top-down inhibitory regulation of the HPA axis and CRH system is impaired in IBS. Our findings may clarify the physiological mechanism underlying stress-induced symptom manifestations in IBS.

## Methods

### Subjects

This study included 28 patients with IBS (14 males; mean age 21.9 ± 2.7 years) diagnosed according to the ROME III criteria^42^ and 34 age-matched, healthy control subjects (18 males, mean age 22.2 ± 2.7 years). All patients with IBS were of the non-constipated subtypes (24 diarrhea-predominant subtype [IBS-D] and 4 mixed subtype [IBS-M]). The subjects were recruited by advertisements and most of the subjects were university students. Each subject underwent a basic evaluation including a medical history review to exclude individual with organic diseases and was given physical examination by a gastroenterology & psychosomatic medicine specialist. None of the subjects had a history of abdominal surgery, endocrinological diseases, or smoking habit. Subjects were asked to complete the following questionnaires: the Japanese version of the IBS Severity Index (Symptom Severity Scale, IBS-SI)^43^, Self-Rating Depression Scale (SDS)^44,45^, State-Trait Anxiety Inventory (STAI)^20,46^, Anxiety Sensitivity Index (ASI)^47^, and the Visceral Sensitivity Index (VSI)^48,49^. Because abuse has been associated with altered HPA axis function in IBS^50^, each subject’s abuse history was assessed by interview based on a questionnaire that was specially validated for a functional gastrointestinal population^51^. More details on these psychometric assessments are provided in supplemental materials.

### Ethics

Subjects were given a description of the study protocol and provided written informed consent for participation. This study was approved by the Ethics Committee of the Tohoku University School of Medicine and was conducted in accordance with the Declaration of Helsinki.

### Study design

The study design is delineated in Fig. 1 (as experiment 1). Subjects fasted overnight and reported for laboratory evaluation at 08:00 the following morning. With the subject in a reclined supine position, cardiac monitoring electrodes were placed, a plastic (Teflon) cannula was inserted into the antecubital vein, and saline was infused at a speed of 0.5 mL/min. Next, a 10-cm, 700-mL capacity polyethylene bag was inserted into the colorectum and taped into place with the distal end of the bag positioned 10 cm from the anal verge. After individual operating pressure (IOP) determination^52^ (details in the supplemental material), the rectal barostat was inflated to IOP for 140 min, and phasic contractions were measured in 5-min intervals. A phasic contraction was defined as a 10% volume reduction (phasic volume event, PVE)^53^ below the IOP lasting 10–60 s. At 20 min after barostat inflation, 2 μg/kg human CRH (hCRH “TANABE” injection, Mitsubishi Tanabe Pharma, Osaka) was dissolved in 2 mL of saline and administered intravenously over 1 min. The dose of CRH was determined based on a previous study^17^. Blood samples for ACTH and cortisol assays were drawn immediately before, and 15, 30, 60, and 120 min after CRH injection. Electrocardiogram (ECG) data were obtained continuously, and the heart rate variability signal analyzed using in-house software in 5-min intervals. The percent power values of the high frequency (HF, 0.15–0.40 Hz) and low frequency (LF, 0.04–0.15 Hz) bands of the Heart rate variability power spectrum were calculated. The HF band was used as a marker of vagal tone, and the LF/HF ratio was used as an indicator of sympathovagal balance^26,27^. More details on the Heart rate variability measurement are provided in the supplemental materials.

### Statistical analyses of endocrine function, heart rate variability, and colonic motility

Data were analyzed using SAS version 9.4 software (SAS Institute, Cary, NC, USA) and expressed as the mean ± SEM unless otherwise stated. A 2-tailed p-value < 0.05 was considered statistically significant. Linear mixed models were used to analyze the time courses of neuroendocrine and autonomic responses to CRH. Time (5 time points for ACTH and cortisol responses, and 28 time bins for autonomic responses) was included as a within-subject categorical independent variable, and group (IBS versus control) and sex were included as between-subject independent variables. Notably, no significant influence of abuse history was found (details in the supplemental material), so abuse history was not included in statistical analyses. In cases of a significant 3-way interaction effect, models were run separately at both levels of the between-subject factors (IBS versus control; females versus males). In cases of a non-significant 3-way interaction effect, the interaction effect was eliminated from the model and the same strategy was applied to non-significant 2-way interactions to generate the most parsimonious, best-fit model while taking into account the number of parameters. In cases of a significant 2-way interaction between group and time or sex and time, the interaction was followed up with a priori planned contrasts using independent sample Student’s t-tests with the stepdown Bonferroni (Holm) correction for multiple comparisons; this strategy was used to compare changes between pre- and post-CRH injection time points between groups and/or between sexes (further details are in the supplemental material).

To normalize the distribution of the colonic motility variable (phasic volume events per 20-min time bin), the value for the pre-infusion time bin was subtracted from the value of each of the 6 post-infusion time bins, resulting in 6 delta variables per subject. These values were then entered into a generalized linear mixed model analysis (gamma distribution with log-link function) with time as a within-subject categorical independent variable, and group and sex as between-subject categorical independent variables. The approach to 3-way and 2-way interaction effects was the same as that described for the other models.

### Study design, brain imaging experiment

The fMRI experiment was designed as 6 runs of 12 trials each, as depicted in Fig. 1 (see experiment 2). Each trial comprised an anticipation-phase period of variable duration (9–18 s), followed by a distention or no distention (18 s) phase and a rating period. Further details on the design are provided in the supplemental material.

All neuroimaging data were acquired on a 3T SIEMENS MAGNETOM TrioTim scanner with a 32-channel head-coil. Functional images were collected using an echo-planar imaging sequence with blood oxygen level-dependent (BOLD) contrast (TR/TE = 3000/30 ms, voxel size = 2.5 * 2.5 * 2.5 mm^3^, flip angle 90, 50 slices) covering the whole brain, including the cerebellum. A total of 240 images were acquired per functional run for a total examination duration of 1 hour and 12 min. A high-resolution structural MRI image was acquired using a 3-dimensional T1-weighted Magnetization Prepared Rapid Acquisition Gradient Echo (3D-MPRAGE) sequence (TR/TE = 2800/2.8 ms, voxel size = 1.0*1.0*1.1 mm^3^) on a different day than the fMRI scan.

We pre-processed and analyzed the fMRI data using SPM8 software (Wellcome Trust Centre for Neuroimaging, UCL). Details of the first (individual) level analysis are described in the supplemental material. At the second (group) level, whole-brain voxel-based analysis was conducted in SPM8 at a voxel-level threshold of p (FWE-corrected) < 0.05, which was adopted for the within-group regression analysis with the individual area under the curve of the ACTH response (ACTH-AUC). The individual ACTH-AUC was calculated from experiment 1, which includes baseline values and is assumed to represent total hormonal output^52^. The association between the brain and ACTH response represents the responsivity of the HPA axis to CRH. Because sex difference^54^ and influence of personality on brain-imaging during gastrointestinal distention have been indicated^55,56^, sex and personality factors were taken into account as nuisance covariates in separate analyses. The first Eigenvariate in each significant cluster from the between-group regression analysis of each subject’s ACTH-AUC was used to visualize the association between brain activity during rectal distention and the ACTH response to CRH. Additionally, ROI analyses with the MarsBar toolbox in SPM8 were conducted for areas involved in HPA axis activation, including the medial (m)PFC, ACC, hippocampus, and amygdala (see Introduction and ref.^9^). ROIs were selected from the Destrieux atlas^57,58^.

### Data availability

The datasets generated during and/or analysed during the current study are available from the corresponding author on reasonable request.

## Electronic supplementary material

Supplementary file
