# Supplementary material for: Altered brain and gut responses to corticotropin-releasing hormone (CRH) in patients with irritable bowel syndrome
Source: Sci Rep. 2017 Sep 29;7:12425. doi: 10.1038/s41598-017-09635-x (PMC5622133; doi:10.1038/s41598-017-09635-x)
Supplement: Supplementary file 1 — Supplementary file [file 41598_2017_9635_MOESM1_ESM.doc]

Supplemental Material

**Altered brain and gut responses to corticotropin-releasing hormone (CRH) in patients with irritable bowel syndrome**

Michiko Kano^1, 2^*, Tomohiko Muratsubaki^2^, Lukas Van Oudenhove^3^, Joe Morishita^2^, Makoto Yoshizawa^4^, Keiji Kono^2^, Mao Yagihashi^2^, Yukari Tanaka^5^, Shunji Mugikura^6^, Patrick Dupont^7^, Huynh Giao Ly^3^, Kei Takase^6^, Motoyori Kanazawa^2^, Shin Fukudo^2^*

^1^Frontier Research Institute for Interdisciplinary Sciences (FRIS), Tohoku University, Japan

^2^Behavioral Medicine, Graduate School of Medicine, Tohoku University, Japan

^3^Laboratory for Brain-Gut Axis Studies (LaBGAS), Translational Research Center for Gastrointestinal Disorders (TARGID), University of Leuven, Leuven, Belgium

^4^Research Division on Advanced Information Technology, Cyberscience Center, Tohoku University, Japan

^5^Department of Integrative Genomics, Tohoku Medical Megabank Organization, Tohoku University, Sendai, Japan

^6^Diagnostic Radiology, Tohoku University Hospital, Japan

^7^Laboratory for Cognitive Neurology, University of Leuven, Leuven, Belgium

**METHODS**

**Psychological assessment**

The Self-Rating Depression Scale (SDS)^1,2^ and State-Trait Anxiety Inventory (STAI)^3,4^ were used to measure depression and anxiety, respectively. The Anxiety Sensitivity Index (ASI) is the most commonly used measure of anxiety sensitivity. This questionnaire measures the following three replicable, lower-order factors: (1) fear of somatic sensations, (2) fear of cognitive dyscontrol, and (3) fear of publicly observable anxiety symptoms^5^. The Visceral Sensitivity Index (VSI) is a self-report measure of the gastrointestinal symptom-specific anxiety^6,7^. Abuse history was assessed with an interview based on an abuse questionnaire^7^, which measured abuse history in two parts: a double 6-item part (yes/no) and double 2-item part (never/seldom/occasionally/often), assessing a history of childhood and adult sexual abuse and physical abuse, respectively^8^. A history of sexual abuse was measured by a positive response to any of the childhood items or to any of the adult items, except for the “exposure of sex organs” item^7^. Physical abuse was considered present when the answer to at least 1 of the physical abuse items was “occasionally” or “often”^7^.

**Study design**

*Sample distention and individual operating pressure (IOP) determination for experiment 1*

At the beginning of the motility testing, sample distention was performed: (1) to ensure that the barostat bag was unfolded, (2) to teach the subject how to use the rating scale to describe colonic sensation intensity, and (3) to decrease anticipatory anxiety. All sensory and motility testing was carried out with the subject lying in a left-lateral position to minimize pressure/bowel compression by overlying body tissues. Subjects were instructed to evaluate the intensities of pain, discomfort, and urgency to defecate during sample distentions or after test distentions on a 0­–10 numerical scale, where 0 indicated no sensation and 10 indicated maximal sensation. During sample distentions, the barostat bag was inflated in a stepwise fashion (4 mm Hg every 15 s) until the subject reported moderate pain (rating of 3). During test distentions, the barostat bag was slowly inflated with 30 mL of air, and the pressure was allowed to equilibrate for 3 min. The average bag pressure during the last 15 s was defined as the IOP, which is the minimum pressure required to overcome mechanical forces for bag inflation with 30 mL of air.

*Heart rate variability (HRV)*

For HRV monitoring, standard Ag/AgCl cutaneous electrodes were applied to the lower left side of the thorax on the mid-axillary line and on the right shoulder over the clavicle. In addition, a round electrode was applied below the subject’s left rib. An electrocardiogram (ECG) was recorded with a sampling frequency of 500 Hz during the examination. The ECG signal was stored on a computer and analyzed with an electrophysiology analysis program (BIMUTAS II; Kissei Comtec, Matsumoto, Japan). Digitized ECG signals were analyzed using in house programs written with MATLAB (Math Works, Natick, MA, USA) and corrected for occasional erroneous recognitions with a semiautomatic procedure that used a parabolic interpolation to increase the accuracy of R-wave recognition and required minimal input from the investigator. A time-frequency analysis based on a Fast Fourier transform using the “spectrogram” function with a moving Hamming window of 37.5 s (128 samples) included in the Signal Processing Toolbox of MATLAB was performed on continuous 2-min segments (epochs) of data in each 5-min period to avoid including artifacts. R-R intervals were calculated, resampled, and interpolated by the program to yield HRV signals for each 5-min block (140 min total) during the colonic phasic motility examination before and after CRH injection. Segments were analyzed using a spectral analysis characterizing heart rate autoregulation by calculating endogenous cardiac activity cycles such as vagus-mediated respiratory sinus arrhythmia. Autonomic influences were discerned according to frequencies in the HRV spectral analysis. The percent power of the high frequency (HF) band of the HRV power spectrum can be used as a marker of vagal tone defined as the percent power in the 0.15–0.40 Hz range, and the low frequency (LF) band in the 0.04–0.15 Hz range reflects sympathetic activity but can include vagal influence. Therefore, the percent power in the HF band as a measure of vagal tone, and the LF/HF ratio as an indicator of sympathovagal balance, were calculated for each segment^9,10^.

*Statistical analyses of endocrine function, heart rate variability, and colonic motility*

Continuous variables were tested for normality using the Shapiro-Wilk test. In cases of non-normality, a logarithmic transformation was used to normalize the distribution. Non-parametric statistical methods were used in cases where normality could not be achieved after the logarithmic transformation.

Demographic characteristics were compared between groups using independent sample Student’s t-tests (or Kruskal-Wallis tests where appropriate) for continuous variables and Pearson χ2-tests (or Fisher exact tests where appropriate) for categorical variables. In linear mixed model analyses, data were modeled by either fitting subject-specific intercepts and linear and quadratic effects of time as a continuous variable (random effects model) or by specifying the most appropriate variance-covariance matrix for the residuals (marginal model) using a random or repeated statement in the proc mixed statement in SAS software (SAS Institute, Cary, NC, USA), respectively. In the latter case, a different variance-covariance matrix was allowed for each level of group and sex based on the observed variance-covariance matrix. The best-fitting model was chosen based on the lowest value of Akaike’s information criterion (AIC). In cases of a non-significant interaction effect, the elimination of 3- and 2-way effects was performed based on an AIC value under maximum likelihood estimation. As the distribution of colonic motility values could not be normalized by applying a logarithmic transformation, values for the pre-infusion time bin were subtracted from those of each post-infusion time bin, resulting in 6 delta variables per subject. In a generalized linear mixed model analysis, we included all 2-way interaction effects, as well as the 3-way interaction effect between group, sex, and time. The data were modeled by fitting subject-specific intercepts using a random statement in the proc glimmix statement in SAS software. A gamma distribution with a log link function provided the best fit based on the lower deviance parameter (-2 res log pseudolikelihood).

Influence of abuse history

Linear mixed models were used to analyze the time courses of adrenocorticotropic hormone (ACTH) and cortisol to assess the influence of abuse history since a previous report found that abuse history increased cortisol response during sigmoidoscopy^8^. Five time points were included as a within-subject categorical independent variable, and abuse history was included as between-subject independent variables. Sex was not included as only 3 males and 6 females had abuse history (among 62 subjects). Group was not included as only 1 control male had abuse history.

*fMRI experimental design*

The day before scanning, subjects were provided low-residue meals and 17 g (13.6%) magnesium citrate, 75 mg sodium picosulfate, and 24 mg sennoside A & B to cleanse the colon. Individual rectal discomfort thresholds were defined prior to the fMRI scanning session. For this purpose, a polyethylene bag tightly fixed to a catheter at both ends was inserted into the colorectum with the distal end of the bag positioned 10 cm from the anal verge and taped into place. The maximum volume of the bag was 700 mL, and the maximum diameter and length of the bag at full inflation were both 10 cm.

*Visceral stimulation*

Rectal distentions were performed using a pressure-controlled barostat system (G&J Electronics Inc., Toronto, Canada). Briefly, rectal perception and discomfort thresholds were determined using the above-described staircase distention method. The retained discomfort threshold was defined as the intra-bag volume eliciting 40–60% discomfort (severe discomfort) with a pain rating of at least 10%. Individually titrated discomfort thresholds were then used throughout the fMRI session to induce rectal discomfort/pain.

*fMRI scanning*

Subjects were placed in a supine position inside the scanner with the rectal balloon inserted but deflated. The balloon was then connected to the barostat located outside of the magnet room via a long, flexible plastic tube that passed through the waveguide. Subjects were asked to pay attention to the visual instructions presented on the screen. A short training sequence (without image acquisition) was performed with a minimum of 1 trial for each sequence type to ensure full comprehension of the protocol and to avoid novelty effects. This short session also allowed the subjects to familiarize themselves with the scanner environment.

The sequence of stimuli during the fMRI experiment was controlled by the dedicated software package Presentation® (NeuroBehavioral Systems, San Francisco, CA, USA). This software was installed on a computer connected to the MRI scanner, barostat, and video-projector and response buttons. Stimulus initiation in each functional run was synchronized with the scanner via detection of the transistor-transistor logic (TTL) pulse produced by the triggering signal from the scanner.

*fMRI trial structure*

Anticipation was triggered by visual cues (symbol presented for 3 s, followed by a fixation point until the end of the distention period): an exclamation point (“!”) indicated a 100% chance that pain would be induced during the subsequent pain period (certain condition), a zero (“0”) indicated that no pain would be induced (safe condition), and a question mark (“?”) indicated a 50% chance that pain would be induced (uncertain condition). Each of the three anticipation conditions (i.e., safe, uncertain, and certain) was repeated 24 times in total. All conditions were randomized within each run, with the constraint that each condition was presented 4 times in each run. During the rating period after distention, visual analogue scale (VAS) ratings (ranging from 0 to 10 with 0 = “no (fear of) pain” and 10 = “worst (fear of) pain ever”) were presented on a screen, and participants had to move a cursor (presented on the screen with a randomized starting position) to the left or right using two keys on a button response box to give their ratings.

*fMRI data analysis*

Pre-processing included spatial realignment to correct for small movements, slice-timing, co-registration of the functional and structural images, segmentation of the structural image, and warping to the Montreal Neurological Institute (MNI) space based on the structural image and on the transformation obtained during the segmentation step. The warping parameters were applied to the functional images (resample using 1.5 x 1.5 x 1.5 mm^3^ voxels), which were then smoothed with a Gaussian isotropic 3D kernel with 8 mm full width at half maximum.

*First (individual) level*

Statistical analyses were conducted with a combined event (anticipation cue) and block (distention/non-distention period) design using a generalized linear model in SPM8. Each condition was modeled as a box-car stimulus function (block conditions) or stick function (event-related conditions), convolved with the canonical hemodynamic response function, and entered into a standard generalized linear model that includes high-pass filtering with a cut-off frequency of 1/128 s to remove low-frequency drifts in the signals. There were 8 regressors of interest corresponding to 3 anticipation types (certain, uncertain, and safe), 4 distention conditions (distention _certain_, distention _uncertain_, non-distention _uncertain_, and non-distention _safe_), and a “rest” condition at the end of each run. The effect of different anticipation contexts on brain activity during rectal distention in IBS have been reported elsewhere^11^. For the purpose of the present study (to test the association of the brain activity during distention and neuroendocrine response), only the contrast between distention following the certain anticipation cue and non-distention periods after safe anticipation cue, were computed for each subject.

**RESULTS**

**Influence of abuse on ACTH and cortisol response to CRH administration**

As there was no influence of abuse on ACTH (main effect of abuse [F_(1, 59)_ = 0.49, p = 0.49 and abuse-by-time interaction effect [F_(4, 236)_ = 0.22, p = 0.92]) or cortisol (main effect of abuse [F_(1, 59)_ = 0.81, p = 0.37] and abuse-by-time interaction [F_(4, 236)_ = 0.42, p = 0.79]) responses, the influence of abuse was not take into account in the analyses on the effect of group and sex on ACTH and cortisol response to CRH administration.

**Influence of visceral hypersensitivity on ACTH response and colonic motility response**

Visceral perception which was defined as intra-bag volume eliciting 40-60% discomfort (severe discomfort) was significantly different between control subjects and IBS patients (249.3 ± 54.8 ml for controls, and 187.8 ± 67.1 ml for IBS, (average ± SD) p<0.001). This visceral sensation threshold was obtained before the fMRI scan and used for colorectal stimulation during fMRI scanning. The visceral sensation threshold was not significantly associated with ACTH-AUC (r=0.05, p=0.8 for controls, r=0.04, p=0.8 for IBS) nor number of PVEs after CRH injection (r=0.04, p=0.8 for controls, r=0.25, p=0.2 for IBS).

**Influence of sex and psychological factors on brain imaging.**

A significantly stronger negative association between ACTH-AUC (ACTH response to CRH) value and brain response during rectal distention was observed in the control group relative to the IBS group. This association was observed in the bilateral ACC (right; x=2, y=39, z=14, cluster size =13, t=5.3, P_FWE-corrected_ =0.02, and left, X=-8, y=38, z=-3, cluster size=9, t=5.24, P_FWE-corrected_ =0.03) and right superior frontal gyrus (x=21, y=38, z=52, cluster size =7, t=5.21, P_FWE-corrected_ =0.03) when sex was added as nuisance covariate. When SDS values were added as a nuisance covariate, the association remained in the bilateral ACC (right; x=2, y=39, z=14, cluster size =30, t=5.3, P_FWE-corrected_ =0.02, and left, X=-8, y=38, z=-3, cluster size=23, t=5.42, P_FWE-corrected_ = 0.02) and right superior frontal gyrus (x=21, y=38, z=52, cluster size =62, t=5.73, P_FWE-corrected_ = 0.008). When STAI values were added as a nuisance covariate, the associations remained in the right ACC (x=2, y=39, z=14, cluster size =17, t=5.4, P_FWE-corrected_ <0.001) and right superior frontal gyrus (x=21, y=38, z=52, cluster size =33, t=5.53, P_FWE-corrected_ =0.01). When ASI values were added as a nuisance covariate, the associations remained in the right ACC (x=2, y=39, z=14, cluster size =50, t=5.53, P_FWE-corrected_ =0.01) and right superior frontal gyrus (x=21, y=38, z=52, cluster size =43, t=5.6, P_FWE-corrected_ =0.01). When VSI values were added as a nuisance covariate, the associations remained in the bilateral ACC (right; x=2, y=39, z=14, cluster size =19, t=5.37, P_FWE-corrected_ =0.02, and left, X=-8, y=38, z=-3, cluster size=1, t=5.12, P_FWE-corrected_ <0.05). Overall, the significantly stronger negative association between ACTH-AUC values and brain responses during rectal distention was observed in the control group relative to the IBS group and remained after taking into account the influence of sex and psychometric factors.

**REFERENCES**

1. Zung, W. W. A Self-Rating Depression Scale. *Arch. Gen. Psychiatry* **12**:63-70 (1965).
2. Fukuda, K. & Kobayashi, S. A. study on a selfrating depression scale (in Japanese). *Shinkeigaku Zasshi* **75**:673-9 (1973).
3. Spielberger, C. D. *et al*. Manual for the State-Trait Anxiety Inventory. Palo Alto, CA: Consulting Psychologists Press, 1983.

4 Nakazato, K. & Shimonaka, Y. The Japanese State-Trait Anxiety Inventory: age and sex differences. *Percept. Mot. Skills* **69**:611-7 (1989).

5 Maruta, T. *et al.* Reliability and validity of the Japanese version of the Anxiety Sensitivity Index. *Compr. Psychiatry* **48**:289-92 (2007).

6 Saigo, T. *et al.* Gastrointestinal specific anxiety in irritable bowel syndrome: validation of the Japanese version of the visceral sensitivity index for university students. *Biopsychosoc. Med* **8**:10 (2014).

7 Labus, J. S. *et al.* The Visceral Sensitivity Index: development and validation of a gastrointestinal symptom-specific anxiety scale. *Aliment. Pharmacol. Ther.* **20**:89-97 (2004).

8 Leserman, J., Drossman, D. A. & Li, Z. The reliability and validity of a sexual and physical abuse history questionnaire in female patients with gastrointestinal disorders. *Behav. Med.* **21**:141-50 (1995).

9 Videlock, E. J. *et al.* Childhood Trauma Is Associated With Hypothalamic-Pituitary-Adrenal Axis Responsiveness in Irritable Bowel Syndrome. *Gastroenterology* **137**:1954-62 (2009).

9 Liu, Q. *et al.* Autonomic functioning in irritable bowel syndrome measured by heart rate variability: a meta-analysis. *J. Dig. Dis.* **14**:638-46 (2013).

10 Elsenbruch, S., Lovallo, W. R. & Orr, W. C. Psychological and physiological responses to postprandial mental stress in women with the irritable bowel syndrome. *Psychosom. Med.* **63**:805-13 (2001)

11 Kano, M. *et al.* Influence of uncertain anticipation on brain responses to aversive rectal distension in subjects with irritable bowel syndrome. *Psychosom. Med.* In press
